# The link between formal thought disorder and social functioning in schizophrenia: A meta-analysis

**DOI:** 10.1192/j.eurpsy.2020.30

**Published:** 2020-03-23

**Authors:** Matthew P. Marggraf, Paul H. Lysaker, Michelle P. Salyers, Kyle S. Minor

**Affiliations:** 1 Department of Psychology, Indiana University Purdue University—Indianapolis, Indianapolis, Indiana, USA; 2 Department of Psychology, Richard L. Roudebush VAMC, Indianapolis, Indiana, USA

**Keywords:** Disorganized, meta-analysis, schizophrenia, social functioning, thought disorder

## Abstract

**Background.:**

Formal thought disorder (FTD) and social functioning impairments are core symptoms of schizophrenia. Although both have been observed for over a century, the strength of the relationship between FTD and social functioning remains unclear. Furthermore, a variety of methodological approaches have been used to assess these constructs—which may contribute to inconsistency in reported associations. This meta-analysis aimed to: (a) systematically test the relationship between FTD and social functioning and (b) determine if the methodology used to assess FTD and/or social functioning moderates this relationship.

**Methods.:**

Following Preferred Reporting Items for Systematic reviews and Meta-analyses (PRISMA) guidelines, a targeted literature search was conducted on studies examining the relationship between FTD and social functioning. Correlations were extracted and used to calculate weighted mean effect sizes using a random effects model.

**Results.:**

A total of 1,478 participants across 13 unique studies were included in this meta-analysis. A small-medium inverse association (*r* = −0.23, *p* < 0.001) was observed between FTD and social functioning. Although heterogeneity analyses produced a significant Q-statistic (*Q* = 52.77, *p* = <0.001), the relationship between FTD and social functioning was not moderated by methodology, study quality, demographic variables, or clinical factors.

**Conclusions.:**

Findings illustrate a negative association between FTD and social functioning. Despite differences in the methodological approach used and type of information assessed, measurement type and clinical factors did not moderate the relationship between FTD and social functioning. Future studies should explore whether other variables, such as cognitive processes (e.g., social cognition), may account for variability in associations between these constructs.

## Introduction

Formal thought disorder (FTD) and social functioning deficits are well-documented as hallmark features of schizophrenia [1,2]. FTD represents a fundamental disruption in the organization and maintenance of goal-directed thought; it affects approximately half of those with schizophrenia [3] and manifests clinically as a core symptom: disorganized speech [4]. Social functioning deficits (e.g., reduced social contact and poor interpersonal skills) are also prevalent in schizophrenia and are an important diagnostic criterion [4]. Both FTD and social deficits persist throughout the disorder and are associated with poor long-term recovery [5–10].

Intuitively, it reasons that those who cannot communicate effectively would also experience difficulties having meaningful social interactions. To this end, schizophrenia researchers have considered how FTD and social functioning are related for decades. Cameron [11] hypothesized that disorganized thought or bizarre speech would be most apparent in social situations and would ultimately lead to withdrawal in social communication. Marengo and colleagues [12] described FTD as being “unique to the particular subject,” “deviant with respect to conventional social norms,” and “frequently hard to understand or empathize with in the context from which the response arose” (p. 498).

Despite longstanding observation, empirical evidence of the relationship between FTD and social functioning deficits in schizophrenia is unclear. Across studies, the magnitude of associations has varied from negligible [13,14] to large [15]. To date, no meta-analysis has synthesized findings to clarify the relationship between FTD and social functioning. This was the primary goal of the current meta-analysis. A secondary goal was to identify possible moderators. In a qualitative review, Roche and colleagues suggested that inconsistencies in conceptualization and measurement of FTD and social functioning may account for variations across studies [1,16]. Thus, measurement approach was tested as a potential moderator in this meta-analysis.

### Conceptualization and measurement of FTD

Bleuler [17] first described FTD as a “loosening of associations” and made a distinction between disordered thought *form* (e.g., derailment and tangentially) and disordered thought *content* (e.g., delusions). FTD is a multifaceted construct, comprised primarily of cognitive and linguistic components [1], and is typically demonstrated behaviorally through speech that is difficult for the listener to understand due to its poor organization and lack of semantic cohesion. In the decades since Bleuler’s original description, two primary methods have been implemented to assess FTD: clinician-rated and trained rater approaches.

#### Clinician-rated measures of FTD

Clinician-rated measures are the most commonly used approach to assess FTD [18–20]. Although differences exist between scales, clinician-rated instruments typically measure FTD using Likert-style ratings based on disorganized speech observed during an interview. Notably, some research using clinician-rated scales has employed a broader conceptualization of FTD, which include a “pure” FTD item, but also include other cognitive or disorganized symptoms. For example, the factor-analytically derived Positive and Negative Syndrome Scale (PANSS [21]) “Disorganized Factor” consists of items assessing pure FTD (conceptual disorganization), behavioral disorganization (e.g., odd/unusual mannerism), and other cognitive symptoms [22]. These factor indices are occasionally used as a proxy for FTD. This may be problematic when examining how FTD relates to social functioning because these additional symptoms likely have their own distinct relationships to social functioning, and thus, may unduly influence reported associations with FTD.

#### Trained rater measures of FTD

Trained rater measures represent another common methodology for assessing FTD. With this approach, specific instances (or behaviors) of FTD are identified, counted, and used to calculate a ratio-level of FTD. Moreover, these measures typically involve sophisticated coding systems that allow for differential weighting of frequency and severity of each instance of FTD. The verbal samples used to assess FTD are typically based on speech elicited in response to a behavioral/stimulus task. The Thought Disorder Index (TDI [23]), for example, assesses FTD in speech samples based on verbatim responses to the Rorschach Test [24]. Trained rater measures generally use standardized scoring systems to assess quantitative and qualitative aspects of FTD and have displayed strong psychometric properties [12,25].

#### Evidence for potential moderation

It is possible that methodological approach will moderate associations between FTD and social functioning. Studies that have examined the degree of convergence between clinician-rated and trained rater measures only report small to moderate associations [13,26–28]. Thus, they may be tapping related, but distinct, aspects of FTD. In addition, trained rater measures have demonstrated more sensitivity to subtle levels of FTD, whereas clinician-rated measures may be designed to capture only gross disturbances [29–31]. If larger associations are found with trained rater measures, it could indicate that subtle FTD disturbances impact social functioning and, thus, further clarify the relationships between these constructs.

### Conceptualization and measurement of social functioning

Establishing a consensus definition of social functioning remains elusive [32–34]. Global definitions often include other functional domains, such as the capacity to function in culturally-defined roles (e.g., worker and student [2]) or include focus on social skills or aspects of social cognition (e.g., social perception [35,36]; see [37,38]). To establish a consistent definition for this meta-analysis, we consulted literature reviews of social functioning measures in schizophrenia [32,34,39,40]. Although no consensus definition emerged, reference to interpersonal relationships and social interactions were included across all definitions and appear to be at the core of social functioning. Therefore, we decided to narrowly define social functioning as an individual’s ability to maintain interpersonal relationships and effectively engage in social interactions. Using this definition, three broad methodological approaches have been used in schizophrenia: self-report, clinician-rated, and performance-based measures.

#### Self-report measures of social functioning

A key distinction of self-report measures, compared to other approaches, is that these measures assess perceived level of social functioning. Generally, self-report measures of social functioning are quick to administer and are useful for obtaining information about the participant’s subjective assessment of their social functioning. However, given the deficits in insight found in schizophrenia, self-report measures may not provide an accurate picture of an individual’s true social functioning.

#### Clinician-rated measures of social functioning

Clinician-rated measures are designed to provide a clinician’s impression of patients’ social functioning. Although this approach still relies on information from patients, clinicians use their expertise to obtain a more nuanced assessment. For example, a patient may report having five close friends but cannot provide their names, illustrating discrepancy between subjective and objective assessments of relationship quality. Notably, clinician-rated measures assess information about social functioning that requires clinical judgment regarding appropriateness or adequate involvement in interpersonal relations with regard to societal standards.

#### Performance-based measures of social functioning

In contrast to other measurement approaches, performance-based measures provide an objective demonstration of social functioning via social interaction. With this approach, participants engage in a simulated social interaction involving another individual (e.g., examiner) and performance is assessed using standardized criteria. For example, the Social Skills Performance Assessment (SSPA [41]), rates participants’ ability to initiate and maintain a conversation in simulated social situations (e.g., meeting a new neighbor) and performance is scored on various criteria (e.g., persistence, engagement, and appropriateness).

#### Evidence for potential moderation

Measurement type may moderate the FTD-social functioning relationship. For example, research has shown that those with FTD underestimate the bizarreness of their speech [42]. Thus, smaller associations between FTD and social functioning may be observed when measured via self-report compared to the more objective clinician-rated and performance-based approaches, as individuals may have limited awareness of the impact that FTD has on their social interactions.

Additionally, unlike clinician-rated and self-report measures, performance-based assessments directly test a person’s ability to interact socially. Disorganized speech arising from FTD may have a greater impact when social functioning is assessed using this measurement approach and could result in larger associations between FTD and social functioning. If measurement type is found to be a significant moderator, it could clarify the impact of FTD on social functioning with regard to subjective perception (i.e., self-report), adequacy of social functioning (i.e., clinician-rated) or actual social interaction (i.e., performance-based).

### Study aims and hypotheses

The overall aim of this meta-analysis is to clarify the relationship between FTD and social functioning in schizophrenia. FTD and social functioning impairments are core features of schizophrenia; they emerge early and often persist throughout the disorder. Thus, a better understanding of their relationship could inform whether there is utility in developing targeted interventions to decrease FTD’s impact (e.g., through adaptive/compensatory strategies or cognitive remediation).

As outlined above, inconsistencies in the operational definition of both FTD and social functioning have contributed to the difficulty in understanding this relationship. We addressed this issue by utilizing narrow definitions of FTD and social functioning, as it is critical to first explore how these variables relate when examining the most fundamental aspects of each construct. The numerous methodological approaches to measuring FTD and social functioning represents another complicating factor in distilling this relationship. Determining the impact of different measurement approaches could further clarify the relationship between these two constructs. Specific aims of this meta-analysis are to:

- Identify the magnitude of the correlation between FTD and social functioning in schizophrenia, which we hypothesize to be negative and in the small to medium range.
- Explore whether measurement type moderates the relationship between FTD and social functioning. Measurement type includes both FTD (clinician-rated and trained rater) and social functioning (self-report, clinician-rated, and performance-based). Study quality, demographic variables (sex and age) and clinical factors (total symptoms, positive symptoms, and negative symptoms) were also examined as potential moderators.

## Methods

### Literature search

The Preferred Reporting Items for Systematic reviews and Meta-analyses (PRISMA [43]) guidelines were used to ensure quality and consistency of meta-analytic reporting. The literature search was executed using three strategies. First, searches were conducted in: Pubmed, PsycINFO, Web of Science, Medline, Dissertation and Thesis (Proquest) and EMBASE, covering journal articles, theses/dissertations, and conference abstracts published up until March 15, 2019. Search terms included a combination of schizophrenia and terms referencing FTD (thought disorder: thought dis* OR disorganized speech: dis*speech) and social functioning (social func* OR quality of life OR interper*). Second, references from pertinent qualitative review articles [1,44] were examined for additional references not detected through the initial search. Third, forward searches of articles directly examining the link between FTD and social functioning [7,15,45–49] were conducted to find additional articles.

### Inclusion and exclusion criteria for study selection

Studies were included in the meta-analysis when they: (a) were available in English; (b) included measures of FTD and social functioning; (c) reported the necessary correlations; and (d) samples consisted of at least 75% with schizophrenia or schizoaffective diagnoses. Authors were contacted if bivariate correlations were not reported and studies were excluded if correlations could not be obtained. A study goal was to examine associations between FTD and social functioning using only the most fundamental aspects of these constructs. Therefore, a priori criteria were established for inclusion as an FTD measure. Specifically, measures had to only assess FTD (e.g., PANSS: Conceptual Disorganization Item) and no other symptoms. Thus, any factor index that assessed FTD in combination with other symptoms was ineligible (e.g., PANSS: Disorganization Factor), unless authors provided correlations between a single FTD item and social functioning.

To be included as a social functioning measure, more than half of the items had to specifically assess interpersonal relationships or social interactions—consistent with our operational definition—or have extractable subscales measuring these domains. This resulted in the exclusion of numerous measures. For example, the Global Assessment of Functioning (GAF) was excluded because it yields a single rating encompassing multiple domains of functioning (i.e., social functioning, occupational functioning, and symptom severity). It also resulted in social cognitive measures (as defined in [38]) being excluded because, although they assess skills necessary for social interaction, they do not examine interaction with another person.

### Coding

Variables from eligible studies were coded using codebooks developed from guidelines suggested by Card [50] and Lipsey and Wilson [51]. Sample-level characteristics of each study were coded, including the percentage of the sample with schizophrenia or schizoaffective disorder, sample size, and demographic information (i.e., average age, sex [percent female], and racial composition, symptom severity). Measurement type and name for FTD and social functioning measures were coded for moderator analyses.

### Effect-size level coding

For each study, raw effect sizes (e.g., correlation coefficient) representing the relationship between FTD and social functioning were extracted. When appropriate, effect sizes were reverse coded so that a negative association always indicated that greater FTD was associated with worse social functioning. Information regarding the type of comparison captured was also coded (e.g., relationship between clinician-rated FTD and self-reported social functioning).

If a study included multiple effect sizes that captured the same type of relationship, effect sizes were averaged and weighted by sample size to reduce bias and avoid violating the assumption of independence [50]. Some studies reported multiple effect sizes capturing unique FTD-social functioning relationships. There was no a priori decision to include one effect size over another; rather, as recommended by Card [50], all effect sizes were retained to maximize the number of comparisons that could be made for categorical moderator analyses. Thus, for the categorical moderator analyses, multiple effect sizes from the same study were included and treated as an independent effect size—which is acceptable when interdependent effects sizes are placed in different subgroups [50]. For the overall meta-analysis, multiple effect sizes from the same study were combined so each study contributed only one effect. Data were coded into Microsoft Excel before being exported to the Statistical Package for the Social Sciences (SPSS) version 24.0, and finally to Comprehensive Meta-Analysis, Version 3 (CMA [52]).

### Meta-analytic method

Descriptive statistics were calculated using SPSS. Mean overall effect sizes were then calculated using CMA. A random effects model was used to account for both within-study and between-study variability [51]. Up to six different effect sizes could be calculated from any study (e.g., clinician-rated FTD–self-reported social functioning, clinician-rated FTD–clinician-rated social functioning). The effect size magnitude was evaluated using Cohen’s [53] suggestions for interpreting correlation effect sizes: *r* = 0.10 (small), *r =* 0.30 (medium), and *r* = 0.50 (large).

Sensitivity analyses and publication bias tests were conducted for the main analysis. A one-study-removed sensitivity analysis was conducted in CMA to identify potential outliers by examining whether the removal of any one effect size from the analysis would substantially change the overall mean effect size [54]. Studies were considered for removal if this change was substantial (e.g., a change from small-medium effect size to a small effect size). Two approaches were employed to assess for the presence of publication bias. First, funnel plots were generated for the main analysis and visually inspected for publication bias. The presence of publication bias was indicated if the plot was not approximately triangular in shape or had an asymmetrical distribution around the mean effect size [54]. Second, publication bias was assessed statistically using the Egger’s regression test [55]. Egger’s tests suggest that publication bias is present when the intercept is significant (*p* < 0.05).

### Heterogeneity and moderator analyses

To examine the presence and extent of heterogeneity, information from both the *Q*-statistic and the *I^2^* index was assessed. A significant *Q-*statistic (*p* < 0.05) indicated the presence of heterogeneity and the *I^2^* index indicated the magnitude of heterogeneity [56]. Moderator analyses were conducted when the *Q*-statistic was significant and the corresponding *I^2^* values were greater than or equal to 25% [57]. Categorical moderators (e.g., measurement type) were categorical and assessed using analysis of variance (ANOVA) analogs provided in CMA and were considered significant when *Q*_between_ was significant (*p* < 0.05) and confidence interval ranges and *I^2^* values were reduced [57]. Continuous moderators (e.g., symptom severity) were assessed using the meta-regression function in CMA using a random-effects model; significant beta weights indicated moderation of the overall effect, and an accompanying decrease in *I^2^* index indicated contribution to the observed heterogeneity.

## Results

### Study selection

**Figure 1.**
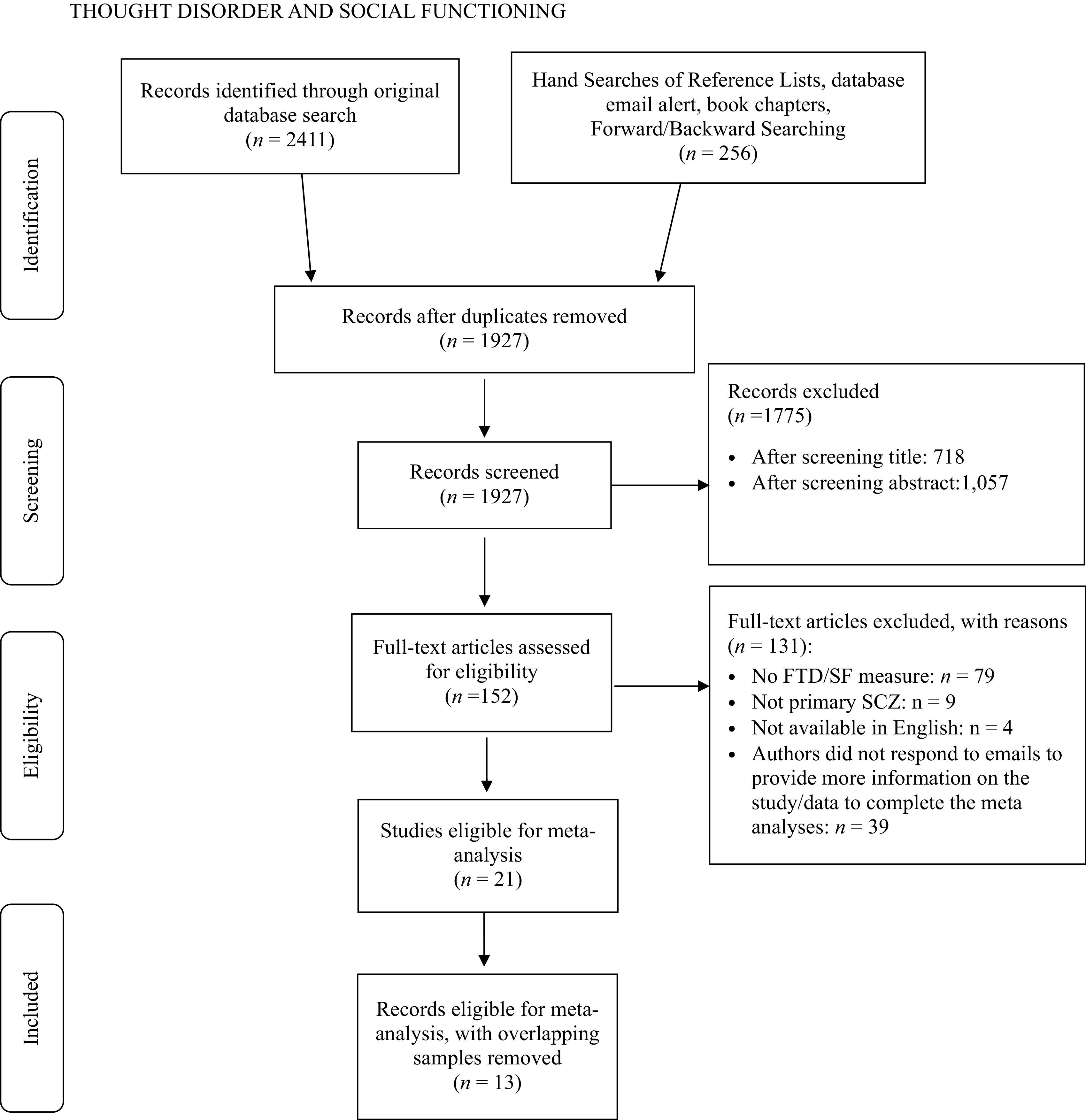
PRISMA study retrieval flow diagram.

Thirteen unique studies were eligible for inclusion in the meta-analysis^1^ (see Figure 1 for PRISMA study retrieval flow diagram). Detailed study characteristics for individual studies are presented in Table 1, and overall aggregated study descriptive statistics are presented in Table 2. Across all eligible studies, a total of 1,478 participants were included.

**Table 1.** Overview of sample characteristics.

Sample characteristics	Mean (SD)	Range	*k*
Mean age	41.0 (7.1)	30.74–56.01	12
Percent female	34.3 (9.2)	24.5–48.6	13
Percent Caucasian	56.9 (23.7)	33.5–95.0	9
Percent African-American	31.6 (23.6)	3.8–66.0	8
Percent diagnosis			
Schizophrenia	84.1 (18.7)	57.0–100.0	10
Schizoaffective	13.7 (16.2)	0–43.0	10
Other psychosis	1.3 (4.8)	0–17.5	13
Mean total symptoms	2.18 (0.21)	1.93–2.40	7
Mean positive symptoms	2.37 (0.31)	1.96–2.75	9
Mean negative symptoms	2.16 (0.45)	1.46–3.00	9
Sample type (*n*, %)			
Peer-reviewed	10 (76.9%)	–	13
Dissertation	2 (15.4%)		13
Other	1 (7.7%)		13
Mean year	2010 (6.4)	1999–2017	13
Mean sample size (total *N* = 1,478)	113.7	40–345	13
Location (*n*, %)			
North America	8 (61.5%)	–	13
Asia	3 (23.1%)	–	13
Australia	1 (7.7%)		13
Europe	1 (7.7%)		13

Abbreviations: Mean total, positive, and negative symptoms, average single item scores on PANSS equivalent symptom rating measure; SD, standard deviation.

**Table 2.** Study-level descriptive statistics for included studies.

Author	Country	*N*	%Fem.	%SCZ/SZA	%Cauc.	Mean age	Mean symps.			FTD measure	Social Fx measure	Association type	*r*
							Tot	Pos	Neg				
Bowie et al. [45]	Canada	90	24.5	100	54.4	–	–	2.60	2.66	TLC	SDS	CR-SR	−0.110
										TLC	SLOF-Soc	CR-CR	−0.180
										TLC	SSPA	CR-PB	−0.350
De Sousa et al. [15]	England	80	27.5	82.5	95.0	39.3	2.33	2.40	2.00	TLC	LSNS	CR-SR	−0.541
										PANSS	LSNS	CR-SR	−0.572
Gilbert [63]	United States	40	30.0	100	40	35.6	2.30	–	–	BPRS: Global FTD	GF: Social	CR-CR	−0.300
										SAPS: FTD	GF: Social	CR-CR	−0.350
										TDI	GF: Social	BB-CR	−0.120
Luther, Lysaker, and Lysaker [57–59]	United States	345	25	95	33.5	45.8	–	2.69	2.27	PANSS	QOL:Inter	CR-CR	−0.001
Moore et al. [64]	United States	72	49	100	43	51.2	2.01	1.99	2.16	EII	SSPA	BB-PB	−0.004
Muralidharan et al. [65]	United States	248	26	100	52.2	56.0	–	1.96	1.98	TLC	SLOF-Soc	CR-CR	−0.216
										TLC	SSPA	CR-PB	−0.139
										TLC	UPSA-Com	CR-PB	−0.282
Nienow [66]	United States	56	25	100	34	41.5	2.40	2.49	1.46	BPRS	AIPSS	CR-PB	−0.220
Racenstein et al. [47]	United States	59	34	100	66	30.74	–	–	–	IPTD-Comp	SCOS-SF	BB-CR	0.100
										IPTD-Prov	SCOS-SF	BB-CR	−0.190
										IPTD-Obj	SCOS-SF	BB-CR	−0.170
Smith et al. [67]	United States	46	37	100	94	39	–	–	–	SAPS:FTD	QOLI-Int	CR-CR	−0.530
Suttajit et al. [68]	Thailand	199	47.7	100	–	37.95	1.93	2.75	1.90	PANSS:	PSP	CR-CR	−0.424
Tan et al. [14]	Australia	54	48.1	100	–	43.35	–	2.01	2.01	PANSS	QOLI:Soc-sbj	CR-SR	−0.090
										PANSS	QOLI:Fam-sbj	CR-SR	0.100
										PANSS	QOLI:Soc-obj	CR-CR	0.030
										PANSS	QOLI:Fam-obj	CR-CR	0.040
Ulas et al. [49]	Turkey	72	39	100	–	35.7	1.94	–	–	TLI-DT	WHOQOL-Soc	BB-CR	−0.182
Yalincetin et al. [69]	Turkey	117	34	100	–	36.02	2.38	2.42	3.00	TLI-DT	PSP	BB-CR	−0.230

Abbreviations: AIPSS, Assessment of Interpersonal Problem Solving Skills; Association type: first abbreviation corresponds to type of FTD measure—BB, behaviorally based; CR, clinician rated; second abbreviation corresponds to type of social functioning measure—CR, clinician rated; PB, performance-based; SR, self-report; BPRS, Global FTD item on the Brief Psychiatric Rating Scale; %Cauc., % of sample that is Caucasian; EII, Ego Impairment Index; Fam-obj, Objective rating on Family item on QOLI; %Fem, % of sample that is female; FTD Meas, name of measure(s) used to assess FTD; GF: Social, Global Functioning: Social Scale; IPTD, Index of Positive Thought Disorder—Comp, Comprehension Subtest—Prov, Proverb Subtest—Obj, Object Sorting Subtest; LSNS, Lubben Social Network Scale; Mean symps., mean symptom; *N*, number of participants included in the study; Neg, mean for negative symptom item; PANSS, Conceptual Disorganization Item on the Positive and Negative Syndrome Scale; Pos, mean for positive symptom item; PSP, Personal and Social Performance Scale; QOL:Inter, Interpersonal Subscale of Quality of Life Scale; QOLI: Fam-subj, Subjective rating for Family item on QOLI; QOLI-Inter, Interpersonal domain on the Quality of Life Interview; QOLI: Soc-subj, Subjective rating for Social item on the Quality of Life Interview; SAPS-FTD, Global item for the FTD subscale of the Scale for the Assessment of Positive Symptoms; SCOS-SF, Strauss Carpenter Outcomes Scale—Social Functioning subscale; %SCZ/SZA, % of sample with schizophrenia or schizoaffective diagnosis; SDS, Sheehan Disability Scale—Social subscale; SLOF-Soc, Specific Level of Functioning Scale—Social Functioning; Soc-sbj, Subjective rating for Social item on QOLI; Soc-obj, Objective rating for Social item on QOLI; Social Fx, name of measure(s) used to assess social functioning; SSPA, Social Skill Performance Assessment; TDI, Thought Disorder Index; TLC, Thought, Language, and Communication Scale; TLI-DT Thought and Language Index—Disorganized Thinking subscale; Tot, mean total symptom item; UPSA-Comm, Communication subtest of the University of California-San Diego Performance-Based Skills Assessment: WHOQOL-Soc, World Health Organization Quality of Life Scale—Social Domain.

A variety of measures were used to assess FTD and social functioning across the 13 studies (see Supplementary Tables S1 and S2 for included measures). For FTD measurement, clinician-rated measures (*k* = 9) were used more frequently than trained rater measures (*k* = 5). For social functioning, clinician-rated measures were the most commonly used (*k* = 11), followed by performance-based measures (*k* = 4) and self-report measures (*k* = 3).

### Sensitivity analyses

Visual inspection of the forest plot indicated heterogeneous effect sizes. The one-study removed analysis and forest plots indicated that the point estimate of the mean effect size did not change drastically with the removal of any study. Therefore, all studies were retained for analyses. Visual examination of funnel plots depicting standardized effect sizes plotted against standard error revealed an overall triangular and approximately symmetrical shape, suggesting that publication bias was not present. The results of the Egger’s regression test (*t*(*11*) = 0.83, *p* = 0.43) also support the conclusion that publication bias was not present in this meta-analytic sample. Forrest and funnel plots available from first author upon request.

### Main analyses

**Figure 2.**
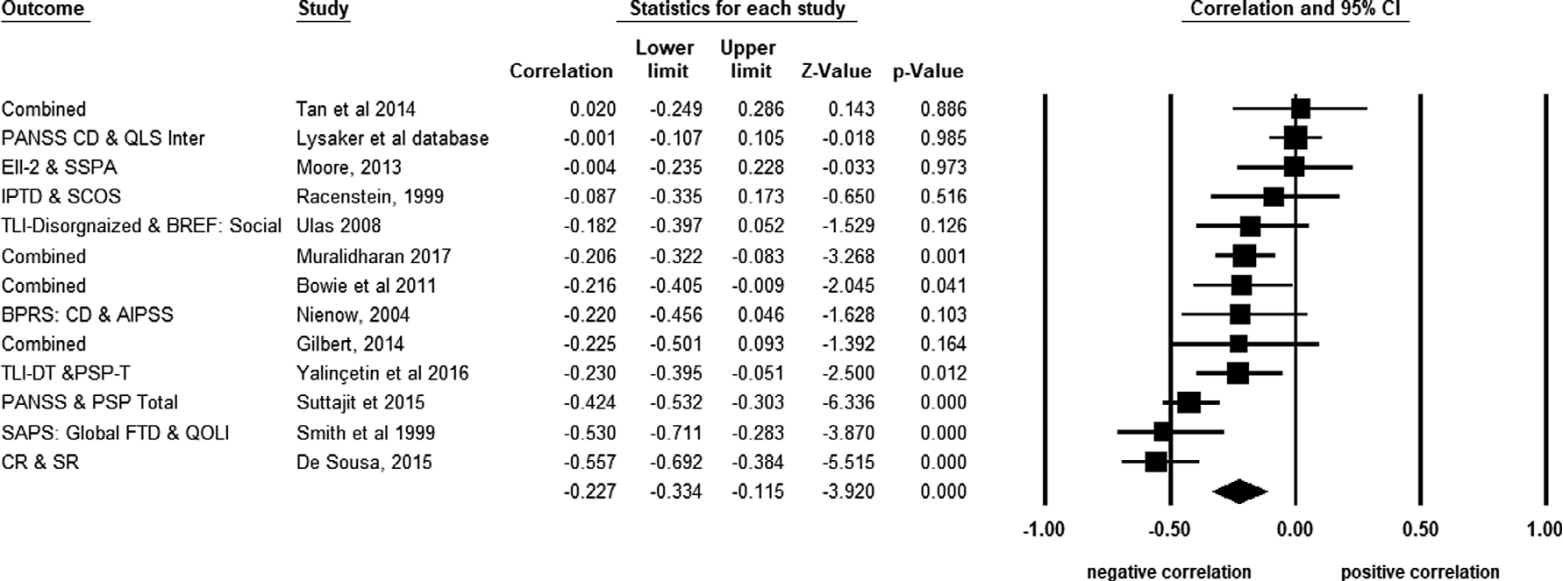
Forest plot of studies included in the meta-analysis examining the relationship between FTD and social functioning (*k* = 13).

Effect sizes from 13 independent samples were included to examine the overall relationship between FTD and social functioning (see Figure 2 for forest plots). Consistent with hypotheses, there was a significant, negative effect size between FTD and social functioning, and the magnitude of the effect size was in the small-medium range (*r* = −0.23, *p* < 0.001, 95% CI [−0.33, −0.12]), indicating greater levels of FTD were associated with worse social functioning (Table 3). Examining the study-level effect sizes, there was a considerable variability in reported associations, with sample-level *r*’s ranging from *r* = −0.56 to *r* = 0.02. Heterogeneity analyses revealed that significant heterogeneity was present (*Q* = 52.77, *p* = *<*0.001), and the extent of heterogeneity (*I^2^* = 77.26) was large, according to guidelines suggested by Huedo-Medina and colleagues [57]. This suggests that results of the main analyses should be interpreted with caution, as the large *I^2^* values indicates the variance is not due to random error and may potentially be explained by moderation analyses [62].

**Table 3.** Summary of mean effect size for the association between FTD and social functioning.

	*k*	ES—*r*	95% CI	*z*	*p*	*Q*	*p*	*I* ^2^
FTD—social functioning	13	–0.23	[–0.33, –0.12]	–3.92	<0.001	52.77	<0.001	77.26

Abbreviations: 95% CI, 95% confidence interval for the mean effect size; ES, sample weighted averaged observed correlation; FTD, formal thought disorder; *I* ^2^, the extent of between-study variability; *k*, number of studies used in the calculation of the mean effect size; *Q*, test for presence of heterogeneity; *Z*, *z*-test for statistical significance of the mean effect size.

### Moderator analyses

Given that significant heterogeneity was present, moderator analyses were conducted on FTD measurement type, social functioning measurement type, study quality, demographic variables, and clinical factors (see Table 4).

**Table 4.** Categorical and continuous moderator analyses.

Categorical moderator	*k*	ES—*r*	95% CI	*z*	*p*	*Q*_bet_, *df* (*p*)	*I^2^*	
FTD measure^a^	14	−0.23	[−0.33, −0.12]	−3.99	<0.001	1.90, 1 (0.168)	75.77	
Clinician-rated	9	−0.28	[−0.40, −0.14]	−3.97	<0.001		83.73	
Behaviorally based	5	−0.13	[−0.31, 0.07]	−1.31	0.192		0.00	
Social function measure^b^	17	−0.21	[−0.30, −0.12]	−4.34	<0.001	1.15, 2 (0.563)	72.63	
Self-report	3	−0.25	[−0.46, −0.02]	−2.10	0.035		87.47	
Clinician-rated	10	−0.21	[−0.33, −0.08]	−3.27	0.001		75.20	
Performance-based	4	−0.20	[−0.38, −0.01]	−1.98	0.048		40.67	
Study quality	13	−0.23	[−0.33, −0.12]	−3.88	<0.001	3.35, 1 (0.067)	77.25	
High	6	−0.28	[−0.44, −0.11]	−3.16	0.002		76.44	
Moderate	7	−0.19	[−0.33, −0.04]	−2.40	0.016		78.72	
Continuous moderator	*k*	*B*	*R* ^2^	SE	95% CI	*z*	*p*	*I^2^* ^c^
%Female	12	−0.21	0.03	0.72	[−1.62, 1.20]	−0.29	0.77	78.03
Age	11	0.01	0.00	0.09	[−0.01, 0.03]	0.84	0.40	80.02
Total symptoms	7	−0.01	0.00	0.01	[−0.03, 0.02]	−0.34	0.74	70.84
Positive symptoms	9	−0.26	0.00	0.27	[−0.79, 0.27]	−0.95	0.34	82.42
Negative symptoms	9	0.07	0.00	0.18	[−0.28, 0.42]	0.42	0.68	82.42

The *p*-value of *Q*_bet_ indicates whether the subgroups are significantly different.

Abbreviations: *B*, regression coefficient; CI, confidence interval; ES, *r* effect size statistic; *I^2^*, extent of between-study variability; *k*, number of studies used in the calculation of the mean effect size; *p*, two-tailed *p*-value associated with the test of statistical significance; *Q*_bet_*,* variance between subgroups; *R^2^*, *R^2^* analogue; SE, standard error, *z*, test for statistical significance of the mean effect size.

a For this analysis, one study [98] contributed effect sizes to both clinician-rated and behaviorally-based subgroups.

b For this analysis three studies contributed multiple effect sizes.

c The *I* ^2^ value might be larger than that of the overall main analyses because not all of the studies were included in the moderator analyses.

#### FTD measurement type

A total of 14 effect sizes were included across clinician-rated (*k* = 9) trained rater measure (*k* = 5) subgroups. Results indicated that FTD measurement type was not a significant moderator, *Q*_between_ = 1.90, *p* = 0.168. At the subgroup level, the average effect size for the clinician-rated measures remained significant, *r* = −0.28 (95% CI [−0.40, −0.14]), *p* < 0.001. However, the average effect size for trained rater measures was not significant, *r* = −0.13 (95% CI [−0.31, .07]), *p* = 0.192.

#### Social functioning measurement type

A total of 17 effect sizes were included in clinician-rated (*k* = 10), performance-based (*k* = 4), and self-report (*k* = 3) measure subgroups. Guidelines suggest a minimum of four effect sizes be included for each subgroup to obtain clinically meaningful results [70]; thus, the self-report subgroup results were not interpreted. Results indicated social functioning measurement type was not a significant moderator, *Q*_between_ = 1.15, *p* = 0.563. At the subgroup level, the average effect sizes remained significant and in the small-medium range, clinician-rated: *r* = −0.21 (95% CI [−0.33, −0.08], *p* = 0.001), and performance-based: *r* = −0.20 (95% CI [−0.38, −0.01], *p* = 0.048).

#### Study quality

Study quality was assessed with the modified Newcastle Ottawa Scale adapted for cross-sectional studies [71], which rated studies on sample selection, assessment, and outcome (high quality: *k* = 6; moderate quality: *k* = 7). The impact of study quality was examined using a categorical moderation analysis, and results indicated that study quality did not significantly moderate this relationship (*p* > 0.05).

#### Continuous moderators

Five continuous moderators were examined using meta-regression analyses: age (*k* = 11), sex (as measured by percent female; *k* = 13), total symptoms (*k* = 7), positive symptoms (*k* = 9), and negative symptoms (*k* = 9). Continuous moderators were run in separate models and none of the continuous moderator Beta weights were significant (all *p*’s > 0.05). Based on the data in this report, average age, sex, and schizophrenia symptoms (total, positive, or negative) do not moderate the relationship between FTD and social functioning.

## Discussion

FTD and social functioning impairments have been considered central features of schizophrenia since its earliest clinical descriptions [17]. Nonetheless, the reported magnitude of associations between these variables have ranged considerably across studies. This is the first time that associations between FTD and social functioning have been synthesized using meta-analytic methods. We observed a significant, inverse relationship between FTD and social functioning of small-medium magnitude. Results were not influenced by measurement type, study quality, demographic variables, or clinical factors. A significantly large amount of heterogeneity was observed, however, suggesting that other variables are moderating the relationship between FTD and social functioning.

The finding that FTD is inversely associated with social functioning has implications for the daily lives of those with schizophrenia. Individuals who have tangential, incoherent, and illogical speech tend to have more difficulty forming and maintaining interpersonal relationships and are less able to effectively engage in social interactions. The small-medium association observed in our study is largely consistent with the magnitude of effects observed when FTD is compared to impairments in other functional domains (e.g., occupational [47,72,73], general community functioning [74,75]). Thus, FTD appears to be associated with an underlying aspect of communication that is necessary for social, occupational, and community functioning.

Although some studies examining FTD and social functioning have used broader conceptualizations, this meta-analysis focused on studies employing pure measures of each construct. As such, our findings can be interpreted as reflecting the overall association between the most fundamental aspects of these constructs. Specifically, for FTD, we included studies that measured FTD only and did not combine measurement of other cognitive domains (e.g., insight and attention) or behavioral disorganization symptoms. Similarly, for social functioning, we included studies that primarily measured the interpersonal relationships and social interaction aspects of social functioning. Given that our primary aim was to clarify the relationship between FTD and social functioning, analyzing the core aspects of these constructs is an important feature of this meta-analysis. Thus, our findings indicate that a small-medium, negative association remains even after removing potentially confounding domains that are included in broader conceptualizations of FTD and social functioning.

Because the effect sizes used in the meta-analysis were based on correlational data, it is not possible to conclude that the presence of greater levels of FTD *cause* impairments in social functioning. However, given the importance of effective communication in human interaction, it has been postulated that greater FTD leads to impairments in social functioning [11,12,76]. For example, Grice [76] has described the “co-operative principle” which proposes that when people engage in communication, there are certain conversational rules or “maxims” that both parties understand should be followed to ensure the meaningful exchange of information (e.g., “be relevant,” “avoid being ambiguous”). Violation of these maxims would be detrimental to social functioning. For instance, if an individual has tangential speech, they will likely discuss topics that are obliquely and ambiguously related to the conversation. This would run counter to the expectations of the conversational partner and leave them confused. In this way, those who have difficulty organizing thoughts and communicating effectively may be viewed by those around them as having poor interpersonal skills, making it difficult to build social relationships.

Conversely, albeit less likely, impaired social functioning may contribute to greater FTD. With limited social interaction, individuals have less opportunity to practice effective communication. When compared to those with more frequent social interactions, these individuals may not receive feedback from others and may be unaware that their speech is difficult to understand. For example, if an individual with FTD received feedback that it is difficult to follow their line of thought, they may attempt to reorganize their thoughts to communicate more efficiently. Perhaps through repeated interactions and subsequent opportunity to practice organizing their thoughts, they may be able to change their thinking patterns.

Contrary to hypotheses, none of the tested variables (measurement type, study quality, demographic [age and sex], or clinical variables [total symptoms, positive symptoms, and negative symptoms]) moderated relationships between FTD and social functioning. This was particularly surprising with measurement type given how methodological approaches assessing both FTD and social functioning differed in the type of information gathered. However, this finding indicates that despite differences in how, for example, social functioning information is gathered—whether through self-report, simulated social interaction, or clinical judgment—the association with FTD is similar. Therefore, a clinician or researcher’s choice of social functioning measurement approach does not appear to unduly influence the relationship with FTD.

Although FTD measurement type was not a significant moderator, an interesting finding did emerge. When examining only trained rater measures, the FTD-social functioning association was reduced and no longer significant. This pattern is consistent with what has been observed in first-episode psychosis, wherein Roche and colleagues [16] found that clinician-rated FTD, but not trained rater FTD, accounted for unique variance in a model predicting social functioning. One potential explanation for these finding centers on the context of speech that is used to assess FTD. Clinician-rated measures assess FTD on speech elicited during clinical interviews, whereas speech elicited in response to task stimuli, (e.g., Gorham Proverbs Task [77]) serve as the basis for ratings with trained rater assessment. It has been argued that speech elicited during behavioral tasks provide limited information about the nature of language and speech in “real” situations [78]. It may be the case that clinical interviews are more reflective of actual social interactions compared to most behavioral tasks.

Although restrictive inclusion criteria for social functioning and FTD measures was a strength of this meta-analysis, it also imposed some limitations. Namely, it resulted in a reduced number of studies eligible for inclusion. Studies were excluded if they assessed FTD using disorganized symptom indices or assessed social functioning with measures that predominantly captured information about other functional domains, symptom severity, or social cognition. Furthermore, an additional methodological approach for assessing FTD—automated assessment—has emerged over the last decade [75,79–82]. Although studies employing automated methods were identified during the literature search [75,83], social functioning measures in these studies failed to meet inclusion criteria. Thus, automated assessment was not included as an FTD methodology in this meta-analysis. Relatedly, our narrow inclusion criteria likely contributed to restricted range in our constructs of interest. In turn, this may have mitigated associations between FTD and social functioning. Another limitation is that the moderating effect of measurement type may have been detected with a more sophisticated analysis examining the FTD-social functioning association at specific levels of each measurement type (i.e., clinician-rated FTD and clinician-rated social functioning versus trained rater FTD and performance-based social functioning). However, given the relatively limited number of effect sizes eligible for inclusion in this study, we did not have a sufficient number of studies to conduct these analyses [70]. Relatedly, there was large heterogeneity in the effects sizes included in this study, indicating that some factor(s) not captured by moderator analyses is influencing the relationship between FTD and social functioning. Finally, there were additional limitations in this study that are common to all meta-analyses (e.g., constraints of the primary studies) [54].

Despite these limitations, our findings have implications for future research. Identification of additional moderators could explain the significant between-study variability of social functioning and FTD observed in this analysis and across the literature. Perhaps underlying cognitive processes could account for some of the heterogeneity. In particular, social cognition appears to be a good candidate, as separate lines of research have found social cognition is linked to both FTD and social functioning. In a meta-analysis, Fett and colleagues [84] found that 5–23% of the variance in functional outcome (including social functioning) was accounted for by social cognition. Medium to large inverse associations have also been demonstrated between FTD and social cognition [85–88]. It is likely that those with higher levels of social cognition may overcome the deleterious effects of FTD and have better social functioning. Conversely, individuals who have high FTD, accompanied by low social cognition, would suffer a “double hit” and may have even worse social functioning. Basic cognition, which has been linked to both FTD [89,90] and functional outcome [5,91–94] could potentially moderate this relationship in a similar fashion.

It may also be useful to investigate how context and setting of speech may affect the FTD-social functioning relationship. Future research should explore methods for assessing FTD in a person’s “real-world” environment and test whether this context would affect the FTD-social functioning relationship. An emerging line of research in healthy populations has employed passive audio-recording devices to capture individual’s speech in natural settings [95,96]. To date, only one study has examined this in schizophrenia-spectrum populations [97]; however, this methodology has the potential to provide unique insight into the impact of FTD on social functioning in people with schizophrenia.

## Conclusion

The results of this meta-analysis indicate that a small-medium relationship exists between social functioning and FTD. Restrictive inclusion criteria were implemented to allow for the examination of this association using core aspects of both constructs. Categorical and continuous moderators (e.g., measurement type) did not account for the large heterogeneity in this study. Future studies should explore whether underlying cognitive processes (e.g., social cognition) or speech settings (e.g., “real” world) could potentially account for the observed heterogeneity.

## Data Availability

The data that support the findings in this meta-analysis can be obtained by contacting the lead author at mpmarggr@iu.edu

## Footnotes

1 It should be noted that one data-set came from a large database that encompassed the overlapping samples of identified studies [58–60]. The full database was the most comprehensive record and avoided the issue of sample overlap and thus, was chosen to be included in the analyses (termed [61]).

## Supplementary material

For supplementary material accompanying this paper visit http://dx.doi.org/10.1192/j.eurpsy.2020.30. click here to view supplementary material
