# Supplementary material for: The link between formal thought disorder and social functioning in schizophrenia: A meta-analysis
Source: Eur Psychiatry. 2020 Mar 23;63(1):e34. doi: 10.1192/j.eurpsy.2020.30 (PMC7355127; doi:10.1192/j.eurpsy.2020.30)
Supplement: Supplementary file 1 [file S0924933820000309sup001.docx]

**Supplementary Table 1**

List of formal thought disorder measures included in meta-analysis.

| Measure | Original article | Description | Speech Sample | FTD score produced | Type |
| --- | --- | --- | --- | --- | --- |
| Expanded Brief Psychiatric Rating Scale (BPRS) | Lukoff et al., 1986 [1] | Symptom Rating Scale. Ratings based on the degree to which thought process are confused, disconnected or disorganized | Clinical Interview | Conceptual Disorganization Item | Clinician-Rated |
| Positive and Negative Syndrome Scale (PANSS) | Kay et al., 1987 [2] | Symptom rating scale that assesses psychiatric symptomatology. Scores based on likert-style rating items. | Clinical Interview | Conceptual Disorganization Item | Clinician-Rated |
| Scale for the Assessment of Positive Symptoms (SAPS) | Andreasen, 1984 [3] | Symptom rating scale which assesses the positive symptoms of schizophrenia including 8 items specifically assessing FTD; ratings are made on Likert scales | Clinical Interview | Global FTD score | Clinician-Rated |
| Thought, Language, and Communication scale (TLC) | Andreasen, 1986 [4] | Assess FTD across 18 different domains, where ratings are made on Likert scales | Unstructured interview | Overall score on TLC | Clinician-Rated |
| Ego Impairment Index (EII) – Derived from the Rorschach Performance Assessment System (RPAS) | Perry, Viglione, & Braff (1992) [5] | Composite measure of thought disturbance. Rating made using a complex coding system which accounts for quantitative and qualitative dimensions of FTD. | Participant responses to Rorschach Ink Blot; verbatim responses recorded | Ego Impairment Index: Total score | Behaviorally-Based |
| Index of Positive Thought Disorder (IPTD) | Marengo et al., 1986 [6] | Scoring system that is applied to verbal responses to assess FTD. Scoring is based on frequency and severity of 11 domains of FTD. Instances of FTD are counted and weighted based on severity. | Responses given to Object Relations Test; Gorham’s Proverbs Test; WAIS-Comprehension; verbatim response recorded | IPTD summary scores from each of three tests. | Behaviorally-Based |
| Thought Disorder Index (TDI) | Johnston & Holzman, 1979 [7]; Solovay et al., 1986 [8] | Assesses 23 aspects of FTD. Each instance of thought disorder is counted and weighted by severity, corrected for response productivity resulting in a percentage multiplied by 100 | Based on transcribed responses from Rorschach Test Items | TDI Total score | Behaviorally- Based |
| Thought and Language Index (TLI) | Liddle et al., 2002 [9] | Assess a variety of language and thought disturbances and produces a disorganized thought subscale. Instances of FTD are counted and weighted based on severity. | Scoring based on transcribed 1-minute responses to each of 8 cards in Thematic Apperception Test | Score on Disorganized Thinking subscale | Behaviorally-Based |

**Supplementary Table 2**

Social functioning measures included in meta-analysis.

| Measure | Original article | Description | Social Functioning score produced | Type |
| --- | --- | --- | --- | --- |
| Lubben Social Network Scale (LSNS) | Lubben, 1988 [10] | Assesses the quality and frequency of contact with family networks, friend networks, and social supports | Total | Self-Report |
| Sheehan Disability Scale (SDS). | Sheehan, 1983 [11] | Self-rated report of impairment and quality of life in social activities, family life and work functioning | Social Subscale | Self-Report |
| Quality of Life Interview (QOLI); Subjective | Lehman (1988) [12] | Assesses patients’ subjective satisfaction with life circumstances, resources, and interpersonal relations (e.g. social relations; family relations) | Social Relations Score  Family Relations Score | Self-Reported |
| Global Functioning: Social | Cornblatt al., 2007 [13] | Interview based measure of social functioning including intimate relationships, close and casual friendships, relationship conflict | Total | Clinician-Rated |
| Heinrich’s Quality of Life (QOL) Scale | Heinrich, et al; 1984 [14] | Quality of Life scale; Interpersonal relations scale assesses relationships family members, close friends, acquaintances, and aspects of social activity (e.g. initiative, withdrawal) | Interpersonal Relations Index | Clinician-Rated |
| Personal and Social Performance (PSP) Scale | Morosini et al., 2000 [15] | Assesses functioning in four domains, three of which are social: social relations, social activities, and aggressive behavior | Total PSP score | Clinician-Rated |
| Quality of Life Interview (QOLI) | Lehman (1988) [12] | Assesses functioning in three domains: life circumstances, resources, and interpersonal relations through structured interview | Interpersonal Relations Score | Clinician-Rated |
| Strauss-Carpenter Outcomes Scale | Strauss & Carpenter, 1972 [16] | Assesses frequency of social contact with friends | Social Index score | Clinician Rated |
| World Health Organization Quality of Life Scale-abbreviated (WHOQOL-BREF) | The WHOQOL Group (1998) [17] | Quality of Life measure; the social relationships domain assesses information about personal relationships, social support and sexual activity | Social Relationships Domain | Clinician Rated |
| Assessment of Interpersonal Problem Solving Skills (AIPSS) | Donahoe et al., 1990 [18] | Role-playing task in which participants identify interpersonal problems in video-taped vignette and act out how they would handle interpersonal problems. | AIPPS overall score | Performance-based |
| Social Skills Performance Assessment | Patterson et al., 2001a [19] | Laboratory-based performance task in which participants are asked to engage in everyday social situations through role play (i.e. meeting a new neighbor and talking to landlord). | SSPA Mean Score | Performance-Based |
| University of California – San Diego Performance Based Skill Assessment | Patterson et al., 2011b [20] | Performance-based measure assessing everyday community functioning. Communication subtest assess performance on role-play task where participant reschedules a doctor’s appointment over the phone. | UPSA-Communication | Performance-Based |

References

[1] Lukoff D, Liberman RP, Nuechterlein KH. Symptom monitoring in the rehabilitation of schizophrenic patients. Schizopr Bull 1986;12:578-603.

[2] Kay SR, Fiszbein A, Opfer LA. The positive and negative syndrome scale (PANSS) for schizophrenia. Schizophr Bull 1987;13:261-276.

[3] Andreasen NC. The Scale for the Assessment of Positive Symptoms (SAPS). Iowa City, IA: The University of Iowa Press; 1984.

[4] Andreasen NC. (1986) Scale for the assessment of thought, language, and communication (TLC). Schizophr Bull 1986;12:473–482.

[5] Perry W, Viglione D, Braff D. The Ego Impairment Index and schizophrenia: A validation study. J Pers Assess 1992;59:165-175.

[6] Marengo JT, Harrow M, Lanin-Kettering I, Wilson A. Evaluating bizarre-idiosyncratic thinking: A comprehensive index of positive thought disorder. Schizophr Bull 1986;12:497-511.

[7] Johnston MH, Holzman PS.  Assessing schizophrenic thinking: A clinical and research instrument for measuring thought disorder. Jossey-Bass; 1979.

[8] Solovay MR, Shenton ME, Gasperetti C, et al. Scoring manual for the Thought Disorder Index. Schizophr Bull 1986;12:483-496.

[9] Liddle PF, Ngan ET, Caissie SL, et al. Thought and Language Index: an instrument for assessing thought and language in schizophrenia. British J Psychiatry 2002;181:326-330.

[10] Lubben JE. Assessing social networks among elderly populations. Fam Community Health 1988;11:42-52.

[11] Sheehan DV. The anxiety disease. Scribner: New York, 1983.

[12] Lehman AF. A quality of life interview for the chronically mentally ill. Evaluation and Program Planning 1988;11:51-62.

[13] Cornblatt BA, Auther AM, Niendam T, et al. Preliminary findings for two new measures of social and role functioning in the prodromal phase of schizophrenia. Schizophr Bull 2007;33:688-702.

[14] Heinrichs DW, Hanlon TE, Carpenter WT. The Quality of Life Scale: An instrument for rating the schizophrenic deficit syndrome. Schizophr Bull 1984;10:388-398.

[15] Morosini PL, Magliano L, Brambilla L, Ugolini S, Pioli R. (2000). Development, reliability and acceptability of a new version of the DSM‐IV Social and Occupational Functioning Assessment Scale (SOFAS) to assess routine social functioning. Acta Psychiatr Scand 2000;101:323-329.

[16] Strauss JS, Carpenter WT. The prediction of outcome in schizophrenia: I. Characteristics of outcome. Arch Gen Psychiatry 1972;27:739-746.

[17] The WHOQOL Group. Development of the World Health Organization WHOQOL-BREF Quality of Life Assessment. Psychological Medicine 1998;28:551–558.

[18] Donahoe CP, Carter MJ, Bloem WD, Hirsch G, Laasi N, Wallace CJ. Assessment of interpersonal problem-solving skills. Psychiatry 1990;53:329-339.

[19] Patterson TL, Moscona S, McKibbin CL, Davidson K, Jeste DV. Social skills performance assessment among older patients with schizophrenia. Schizophr Res 2001;48:351-360.

[20] Patterson TL, Goldman S, McKibbin CL, Hughs T, Jeste DV. UCSD Performance-Based Skills Assessment: development of a new measure of everyday functioning for severely mentally ill adults. Schizophr Bull 2001;27:235-45.
